# Draft Genome Sequence of *Staphylococcus succinus* Strain CSM-77, a Moderately Halophilic Bacterium Isolated from a Triassic Salt Mine

**DOI:** 10.1128/genomeA.00532-16

**Published:** 2016-06-09

**Authors:** Julianne Megaw, Brendan F. Gilmore

**Affiliations:** School of Pharmacy, Queen’s University Belfast, Belfast, United Kingdom

## Abstract

Here, we report the draft genome sequence of *Staphylococcus succinus* strain CSM-77. This moderately halophilic bacterium was isolated from the surface of a halite sample obtained from a Triassic salt mine.

## GENOME ANNOUNCEMENT

*Staphylococcus succinus* is a Gram-positive coccoid bacterium first identified in 1998 after its isolation from Dominican amber (1). This species has since been isolated from diverse environments, including cheese, dry or fermented meat products, human clinical specimens, and the Dead Sea (2 – 6). *S. succinus* strain CSM-77 was isolated from the surface of a fragment of Triassic halite, collected from Kilroot salt mine, Carrickfergus, Northern Ireland, on Sehgal-Gibbons agar (7) containing 15% NaCl, at 28°C. The strain was maintained on Luria-Bertani (LB) agar and showed optimal growth when this medium was supplemented with 5% NaCl, indicating that it is a moderate halophile. It showed 100% 16S sequence similarity with the type strain *S. succinus* AMG-D1 (1).

Genomic DNA was extracted from a culture freshly grown in LB broth with 5% NaCl using a GenElute bacterial genomic DNA kit (Sigma-Aldrich, United Kingdom), following the protocol for Gram-positive bacteria. Whole-genome sequencing was performed by MR DNA (Shallowater, TX, USA), using the Illumina MiSeq platform. The sequence reads were assembled *de novo* by MR DNA using the NGen DNA assembly software by DNAStar, Inc.

The assembled genome contained 11 contigs (longest 839,952 bp), with a total size of 2,802,639 bp, and a GC content of 32.95%. Annotation in RAST (8) revealed 413 subsystems, 2,654 coding sequences, and 72 RNAs. Several genes were identified that were associated with resistance to heavy metals and toxic compounds (copper, cobalt, zinc, cadmium, mercury, arsenic, and chromium), antibiotic resistance (teicoplanin, fluoroquinolones, β-lactams, and multidrug resistance genes), as well as bacteriocin production. The genome was also examined using AntiSMASH (9) and seven secondary metabolite biosynthetic gene clusters were identified, including four microcins.

### Nucleotide sequence accession numbers.

This whole-genome shotgun project has been deposited at DDBJ/ENA/GenBank under the accession number LUJH00000000. The version described in this paper is the first version, LUJH01000000.
